# Protective effect of transgenic expression of porcine heat shock protein 70 on hypothalamic ischemic and oxidative damage in a mouse model of heatstroke

**DOI:** 10.1186/1471-2202-10-111

**Published:** 2009-09-03

**Authors:** Zhih-Cherng Chen, Wen-Shian Wu, Mao-Tsun Lin, Chuan-Chih Hsu

**Affiliations:** 1 Section of General Medicine, Chi Mei Medical Center, Tainan, Taiwan, Republic of China; 2 Chung Hwa University of Medical Technology, Tainan, Taiwan, Republic of China; 3 Department of Cardiology, Chi Mei Medical Center, Tainan, Taiwan, Republic of China; 4 Department of Medical Research, Chi Mei Medical Center, Tainan, Taiwan, Republic of China; 5 Department of Surgery, Chi Mei Medical Center, Tainan, Taiwan, Republic of China

## Abstract

**Background:**

Transgenic mice have been used to examine the role of heat shock protein (HSP)72 in experimental heatstroke. Transgenic mice that were heterozygous for a porcine HSP70β gene ([+] HSP72) and transgene-negative littermate controls ([-] HSP72), under pentobarbital sodium anesthesia, were subjected to heat stress to induce heatstroke. It was found that the overexpression of HSP72 in multiple organs improved survival during heatstroke by reducing hypotension and cerebral ischemia and damage in mice. Herein we attempted to further assess the effect of heat exposure on thermoregulatory function, hypothalamic integration, and survival in unrestrained, unanesthetized [+]HSP72 and compare with those of [-]HSP72. In this research with the transgenic mice, we first conducted several biochemical, physiologic and histological determinations and then investigated the beneficial effects of HSP72 overexpression on the identified hypothalamic deficits, thermoregulatory dysfunction, and mortality during heatstroke.

**Results:**

We report here that when [-]HSP72 mice underwent heat stress (ambient temperature 42.4°C for 1 h), the fraction survival and core temperature at 4 h after heat stress were found to be 0 of 12 and 34.2°C ± 0.4°C, respectively. Mice that survived to day 4 after heat stress were considered as survivors. In [+]HSP72 mice, when exposed to the same heat treatment, both fraction survival and core temperature values were significantly increased to new values of 12/12 and 37.4°C ± 0.3°C, respectively. Compared to [-]HSP mice, [+]HSP72 mice displayed lower hypothalamic values of cellular ischemia (e.g., glutamate and lactate-to-pyruvate ratio) and damage (e.g., glycerol) markers, pro-oxidant enzymes (e.g., lipid peroxidation and glutathione oxidation), pro-inflammatory cytokines (e.g., interleukin-1beta and tumor necrosis factor-alpha), and neuronal damage score evaluated 4 h after heat stress. In contrast, [+]HSP72 mice had higher hypothalamic values of antioxidant defences (e.g., glutathione peroxidase and glutathione reductase), ATP, and HSP72 expression.

**Conclusion:**

This study indicates that HSP72 overexpression appears to be critical to the development of thermotolerance and protection from heat-induced hypothalamic ischemic and oxidative damage.

## Background

Recently, it has been shown that heat stress causes thermoregulatory deficits in unrestrained, unanesthetized mice [1,2]. Heat stressed mice become hypothermic when exposed to room temperature. The heat stress-induced hypothermia in mice may result from ischemic damage to their hypothalamus (the essential thermoregulatory center in brain).

It has also been reported that healthy volunteers display higher serum levels of heat shock protein (HSP) 72 than those of heatstroke patient [3]. Increased serum levels of HSP72 appears to correlate with heat tolerance. This hypothesis is further supported by experimental studies. For example, sublethal heat shock pretreatment 16 h before initiation of heatstroke, in addition to increasing HSP72 in multiple organs, conferred significant protection against heatstroke-induced circulatory shock and cerebral ischemia and oxidative damage [4] in anesthetized rats. Both L-arginine [1,5] and glutamine [6] have also been shown to increase the expression of HSP72 in multiorgans and reduce mortality in a rat model of heatstroke. However, it is not known whether the heat-induced hypothalamic ischemia and oxidative damage, thermoregulatory dysfunction and mortality can be affected by HSP72 overexpression in transgenic mice.

To deal with the question, in this research with the transgenic mice, we first conducted several biochemical, physiological, and histological determination and then investigated the beneficial effects of HSP72 overexpression on the identified hypothalamic deficits, thermoregulatory dysfunction, and mortality during heatstroke [2].

## Results

### HSP72 overexpression in the hypothalamus of [+]HSP72 mice

**Figure 1:**
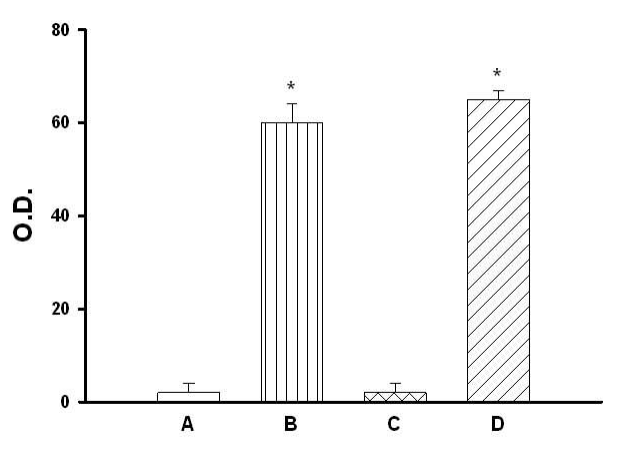
**O.D. values of protein assay by the densitometer for hypothalamic tissues obtained from non-heated [-]HSP72 mice (A), non-heated [+]HSP72 mice (B), heated [-]HSP72 mice (C), and heated [+]HSP72 mice (D)**. The animals were killed 4 h post whole body heating for heated group or the equivalent time period for non-heated group. Data are expressed as means ± S.E.M. for 8 mice per group. *P < 0.01 compared with non-heated [-]HSP72 mice or heated [-]HSP72 mice.

The O.D. values of protein assay by the densitometer for hypothalamic tissues obtained from different groups are summarized in Figure 1. The animals were killed 4 h post whole body heating (WBH) for heated groups or the equivalent time period for non-heated groups. Western analyses revealed that both heated and non-heated [+]HSP72 mice had higher hypothalamic expression of HSP72 than those of both heated and non-heated [-]HSP72 mice (P < 0.001).

### HSP72 overexpression attenuates heat-induced lethality and hypothermia

**Figure 2:**
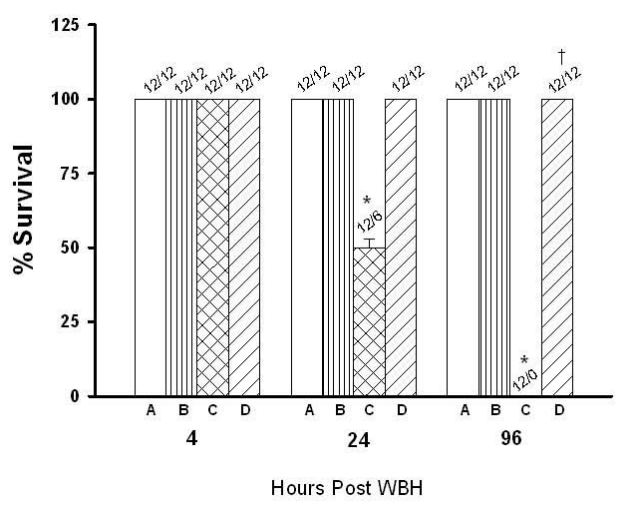
**Percent survival values for non-heated [-]HSP72 mice (A), non-heated [+]HSP72 mice (B) heated [-]HSP72 mice (C), and heated [+]HSP72 mice 4-96 hours post whole body heating**. Data are expressed as means ± S.E.M. for 12 mice per group. *P < 0.001 compared with non-heated [-]HSP72 mice; ^†^P < 0.001 compared with heated [-]HSP72 mice.

As summarized in Figure 2, as compared to non-heated [-]HSP72 mice, the percent survival values of heated [-]HSP72 mice were significantly lower when evaluated at both 24 h and 96 h post WBH (50% and 0%, respectively; P < 0.001). The heat-induced lethality was completely abolished by overexpression of HSP72 in the hypothalamus of [+]HSP72 mice (100%, P < 0.001). Four hours after heat stress, the core temperature values of heated [-]HSP72 mice were significantly lower than those of non-heated [-]HSP72 mice kept at a normal ambient temperature (26°C) (P < 0.001; Table 1). Again, the heat-induced hypothermia was completely abolished by overexpression of HSP72 in the hypothalamus (37.4°C; P < 0.001).

**Table 1:** Core temperature values obtained from non-heated [-]HSP72 ([-]HSP72-N) mice, non-heated [+]HSP72 ([+]HSP72-N) mice, heated [-]HSP72 ([-]HSP72-H) mice, and heated [+]HSP72 ([+]HSP72-H) mice.

**Treatment Groups**	**Core temperature (°C)**	***P *values**
[-]HSP72-N mice	37.5 ± 0.2	
[+]HSP72-N mice	37.3 ± 0.3	
[-]HSP72-H mice	34.2 ± 0.4	^a^P < 0.001
[+]HSP72-H mice	37.4 ± 0.4	^b^P < 0.001

Core temperatures were measured 4 h post whole body heating for heated groups or the equivalent time period for non-heated groups. ^a^Compared with non-heated groups; ^b^Compared with [-]HSP72-H mice. Data are means ± S.E.M. of 12 mice per group.

### HSP72 overexpression reduces heat-induced increased levels of glutamate, glycerol, lactate-to-pyruvate ratio, dihydroxybenzoic acid (DHBAs) and nitrite in the hypothalamus

Table 2 summarizes the effects of heat exposure (42.4°C for 1 h) on cellular levels of glutamate, lactate-to-pyruvate ratio, glycerol, nitrite, and DHBA in the hypothalamus in both [-] and [+]HSP72 mice. As compared with non-heated controls, the heated [-]HSP mice had higher levels of glutamate, lactate-to-pyruvate ratio, glycerol, nitrite, and DHBA in the hypothalamus (P < 0.05). However, as compared to those of heated [-]HSP72 mice, heated [+]HSP72 mice had lower levels of all these parameters in the hypothalamus (P < 0.05).

**Table 2:** The concentrations of glutamate, glycerol, lactate/pyruvate ratio, DHBA, and nitrite in the extracellular levels of the hypothalamus obtained from non-heated [-]HSP72 ([-]HSP72-N) mice, non-heated [+]HSP72 ([+]HSP72-N) mice, heated [-]HSP72 ([-]HSP72-H) mice, and heated [+]HSP72 ([+]HSP72-H) mice.

**Treatment Groups**	**Glutamate (% of baseline)**	**Lactate/pyruvate ratio**	**Glycerol (% of baseline)**	**Nitrite (μM)**	**DHBA (% of baseline)**
[-]HSP72-N	100 ± 3	9 ± 1	102 ± 3	14 ± 2	100 ± 5
[+]HSP72-N	100 ± 2	8 ± 2	101 ± 2	15 ± 3	101 ± 6
[-]HSP72-H	178 ± 9^a^	213 ± 14^a^	156 ± 7^a^	98 ± 9^a^	142 ± 7^a^
[+]HSP72-H	108 ± 6^b^	25 ± 4^b^	21 ± 3^b^	26 ± 4^b^	109 ± 6^b^

Samples were measured 4 h post whole body heating or the equivalent time period for non-heated groups. ^a^Compared with non-heated groups (P < 0.05); ^b^Compared with [-]HSP72-H group (P < 0.05). Data are means ± S.E.M. of 12 mice per group.

### HSP72 overexpression attenuates heat-induced hypothalamic neuronal damage

**Figure 3:**
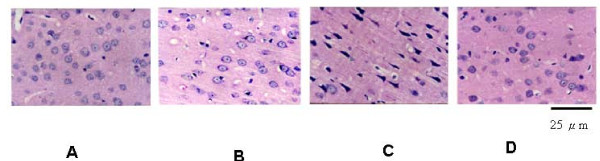
**Photomicrographs of hematoxylin-eosin staining of the hypothalamus of a non-heated [-]HSP72 mouse (A), a non-heated [+]HSP72 mouse (B), a heated [-]HSP72 mouse (C), and a heated [+]HSP72 mouse (D)**. The animals were killed 4 h post whole body heating for heated group or the equivalent time period for non-heated group. (Original magnification ~400).

Table 3 summarizes the effect of heat exposure (42.4°C for 1 h) on the neuronal damage scores of the hypothalamus from heated or non-heated [-]HSP72 and [+]HSP72 mice. For determination of neuronal damage, animals were killed 4 h after heat exposure. The heated [-]HSP70 mice exhibited cell shrinkage and pyknosis of the nucleus in their hypothalamus 4 h after the termination of heat stress. However, the heat-induced neuronal damage in the hypothalamus were lower in a heated [+]HSP70 mice (Table 3; P < 0.05). A typical example is depicted in Figure 3.

**Table 3:** The neuronal damage score values of the hypothalamus obtained from non-heated [-]HSP72 ([-]HSP72-N) mice, non-heated [+]HSP72 ([+]HSP72-N) mice, heated [-]HSP72 ([-]HSP72-H) mice, and heated [+]HSP72 ([+]HSP72-H) mice.

**Treatment Groups**	**Neuronal damage score (0-3)**
[-]HSP72-N	0 (0, 0)
[+]HSP72-N	0 (0, 0)
[-]HSP72-H	2 (2, 2)^a^
[+]HSP72-H	0.75 (0, 0.75)^b^

For determination of neuronal damage scores, heated groups were killed 4 h post whole body heating or the equivalent time period for non-heated groups. ^a^Compared with non-heated groups (P < 0.05); ^b^Compared with [-]HSP72-H (P < 0.05). Data are medians from 12 mice per group, followed by Q1 and Q3 in parentheses.

### HSP72 over-expression attenuates heat-induced hypothalamic oxidative stress and ATP depletion

As shown in both Table 4 and Table 5, 4 h after the start of heat stress, the hypothalamic levels of malondialdehyde (MDA), oxidized-form glutathione (GSSG) and GSSG/reduced-form glutathione (GSH) in heated [-]HSP72-mice were significantly higher than those of non-heated [-]HSP72 mice. In contrast, the hypothalamic levels of GSH, glutathione peroxidase, glutathione reductase, and ATP of heated [-]HSP72-mice were significantly higher than those of non-heated [-]HSP72 mice (P < 0.05). The heat-induced increased levels of malondialdehyde, GSSG, and GSSG/GSH as well as the decreased levels of GSH, glutathione peroxidase, glutathione reductase, and ATP in the hypothalamus was significantly reduced by overexpression of hypothalamic HSP72 in [+]HSP72 mice.

**Table 4:** The hypothalamic concentrations of malondialdehyde, oxidative-form glutathione (GSSG), reduced-form glutathione (GSH), and GSSG/GSH ratio obtained from non-heated [-]HSP72 ([-]HSP72-N) mice, non-heated [+]HSP72 ([+]HSP72-N) mice, heated [-]HSP72 ([-]HSP72-H) mice, and heated [+]HSP72 ([+]HSP72-H) mice.

**Treatment Groups**	**Malondialdehyde (n mol/mg protein)**	**GSSG** **(pmol/μg prot.)**	**GSH** **(pmol/μg prot.)**	**GSSG/GSH**
[-]HSP72-N	4.5 ± 0.3	2.2 ± 0.2	3.9 ± 0.3	0.56 ± 0.15
[+]HSP72-N	4.6 ± 0.3	2.4 ± 0.3	3.8 ± 0.4	0.63 ± 0.22
[-]HSP72-H	9.9 ± 0.8^a^	3.6 ± 0.2^a^	2.0 ± 0.4^a^	1.80 ± 0.31^a^
[+]HSP72-H	4.8 ± 0.4^b^	2.5 ± 0.3^b^	4.2 ± 0.4^b^	0.59 ± 0.18^b^

Samples were measured 4 h post whole body heating for heated groups or the equivalent time period for the non-heated groups. ^a^Compared with non-heated groups (P < 0.05); ^b^Compared with [-]HSP72-H group (P < 0.05). Data are means ± S.E.M. of 12 mice per group.

**Table 5:** Hypothalamic levels of glutathione peroxidase, glutathione reductase and ATP obtained from non-heated [-]HSP72 ([-]HSP-N) mice, non-heated [+]HSP72 ([+]HSP72-N) mice, heated [-]HSP72 ([-]HSP72-H) mice, and heated [+]HSP72 ([+]HSP72-H) mice.

**Treatment Groups**	**Glutathione peroxidase** **(mU/mg protein)**	**Glutathione reductase** **(mU/mg protein)**	**ATP** **(pmoles/mg wet wt)**
[-]HSP72-N	345 ± 46	177 ± 18	38 ± 6
[+]HSP72-N	362 ± 51	165 ± 17	44 ± 5
[-]HSP72-H	108 ± 29^a^	99 ± 14^a^	16 ± 4^a^
[+]HSP72-H	367 ± 58^b^	166 ± 21^b^	40 ± 7^b^

Samples were measured 4 h post whole body heating for heated groups or the equivalent time period for the non-heated groups. ^a^Compared with non-heated groups (P < 0.05); ^b^Compared with [-]HSP72-H group (P < 0.05). Data are means ± S.E.M. of 12 mice per group.

### HSP72 overexpression attenuates heat-induced overproduction of both interleukin-1β (IL-1β) and tumor necrosis factor-α (TNF-α)

As shown in Table 6, 4 h post whole body heating, hypothalamic levels of both IL-1β and TNF-α were increased in [-]HSP72 heated mice. Again, heat-induced overproduction of both IL-1β and TNF-α were significantly reduced in [+]HSP72 heated mice (P < 0.05). In both heated and non-heated [-]HSP72 mice, hypothalamic levels of interleukin-10 (IL-10) were maintained at a negligible levels (Table 6). However, hypothalamic levels of IL-10 were greatly elevated in [+]HSP72 non-heated or heated mice, as compared to those of [-]HSP72 mice (P < 0.05).

**Table 6:** Hypothalamic levels of TNF-α, IL-1β, and IL-10 obtained from non-heated [-]HSP72 ([-]HSP72-N) mice, non-heated [+]HSP72 ([+]HSP72-N) mice, heated [-]HSP72 ([-]HSP72-H) mice, and heated [+]HSP72 ([+]HSP72-H) mice.

**Treatment Groups**	**TNF-α (pg/g)**	**IL-1β (pg/g)**	**IL-10 (pg/g)**
[-]HSP72-N	7 ± 2	5 ± 2	2 ± 1
[+]HSP72-N	6 ± 2	4 ± 2	93 ± 7
[-]HSP72-H	173 ± 21^a^	448 ± 47^a^	5 ± 3^a^
[+]HSP72-H	44 ± 5^b^	59 ± 6^b^	101 ± 8^b^

Samples were measured 4 h post whole body heating for heated groups or the equivalent time period for the non-heated groups. ^a^Compared with non-heated groups (P < 0.05); ^b^Compared with [-]HSP72 group (P < 0.05). Data are means ± S.E.M. of 12 mice per group.

## Discussion

It has been shown that severe heat exposure causes profound thermoregulatory dysfunction (e.g., heat stressed animals had lower core temperature, e.g., 34°C when exposed to room temperature 26°C) and mortality [1,2]. Our data further demonstrated that the heat-induced thermoregulatory dysfunction and mortality may be resulted from ischemia and oxidative damage to their hypothalamus (the essential thermoregulatory center in brain) in the mouse. The latate-to-pyruvate ratio is a well-known marker of cellular ischemia, whereas glycerol is a marker of how severly cells are affected by ongoing pathology [7]. Compared to normothermic control, heatstroke mice had significantly higher levels of cellular ischemia (e.g., glutamate and lactate-to-pyruvate ratio) and damage (e.g., glycerol) markers in their hypothalamus [8-13]. These heatstroke mice also displayed significantly higher hypothalamic levels of reactive oxygen species (e.g., DHBA and superoxide anions), lipid peroxidation (e.g., MDA), and enzymatic pro-oxidants (e.g., GSSG/GSH), but lower hypothalamic levels of enzymatic anti-oxidant defences [e.g., activity of both glutathione reductase (GR) and glutathione peroxidase (GPx)] during heatstroke. Increased production of reactive oxygen species has been reported to be directly involved in oxidative damage with cellular macromolecules in ischemic brain tissues, which lead to cell death [14]. Probably, the most striking finding of the present study is that overexpression of HSP72 in the hypothalamus significantly protects against the heat-induced hypothalamic ischemia and oxidative damage, thermoregulatory dysfunction, and mortality. The present results are in part consistent with several previous experiments. For example, L-arginine administration to heat-stressed mice [3,6] or glutamine administration to heat-stressed rats [15] increases the expression of HSP72 and contributes to the protective effect of these drugs in rodents subjected to heatstroke. Sublethal heat shock pretreatment 16 h before initiation of heatstroke, in addition to increasing brain HSP72 levels, conferred significant protection against heatstroke-induced brain oxidative damage in anesthetized rats [4].

In the rodents, environmental heat stress increases cutaneous blood flow and metabolism and progressively decreases splanchnic blood flow. Reduced splanchnic ischemia coupled with increased metabolism causes hyperthermia and cellular ischemia and hypoxia in peripheral organs [16]. After the onset of heatstroke, loss of blood flow to the brain (arising from both arterial hypotension and intracranial hypertension) results in neuronal injury due to both the cessation of blood flow leading to oxygen and nutrient deprivation and the initiation of a cascade of secondary mechanism [17,18]. Indeed, it has been shown that nitric oxide levels in multiple organs are elevated in heatstroke rats [5,17-19]. Heat-induced multiple organ dysfunction can be greatly reduced by inducible and neuronal nitric oxide synthase inhibitors. In the present study, when exposed the unanesthetized and unrestrained mice to high ambient temperatures, overproduction of nitric oxide in their hypothalami was noted after the onset of heatstroke. Heat-induced overproduction of nitric oxide in the hypothalamus was greatly reduced by hypothalamic HSP72 overexpression in [+]HSP mice. It should be stressed that, in the present study, we measured only the hypothalamic levels of nitric oxide. It is reasonable to assume that the similar outcome can be seen in other organs. Therefore, based on these findings, we propose that overexpression of HSP72 in multiple organs may attenuate heatstroke reactions by reducing heat-induced overproduction of nitric oxide.

In anesthetized rats, overproduction of both IL-1 and TNF-α in both the peripheral blood stream and the brain occurred during heatstroke [20,21]. The same phenomenon was also observed in unanesthetized, unrestrained mice attendanted with heatstroke, as shown in the present results. Overexpression of HSP72 in [+]HSP72 mice before the initiation of heat stress significantly prevented the heat-induced overproduction of proinflammatory cytokines in brain, hypotension, and animal death. In particular, overproduction of IL-10 in brain was evoked in heated [+]HSP72 mice. This implies that HSP72 overexpression may improve heat tolerance by increasing IL-10 but decreasing TNF-α and IL-1β production in multiple organs. The contention is supported by many investigators. For example, IL-10 knockout mice have an increased extent of inflammation illness [22] and higher mortality rates after experimental sepsis [23]. Exogenous administration of recombinant IL-10 protects mice from lethal endotoxemia by reducing TNF-α release [23]. In endotoxemic mice, neutralization of endogenously produced IL-10 results in an increased production of proinflammatory cytokines and enhanced mortality [24].

At the cellular level, failing energy metabolism and ATP depletion are the earliest cell-damaging factors of ischemic insult. In vivo, severe depletion of ATP causes proteotoxic stress that leads to dysfunction, destabilization and aggregation of many proteins [25,26]. The present results further found that prior HSP72 overexpression in [+]HSP72 mice significantly shields against heat-induced reactions by maintaining appropriate levels of ATP in hypothalamic tissues.

Our previous outcomes [27] have identified that, in anesthetized mice, HSP72 overexpression in multiple organs preserves against hypotension and striatal ischemic injury during heatstroke. In the current findings, the hypothalamic HSP72 overexpression protects unanesthetized, unrestrained mice from heatstroke-induced death, thermoregulatory deficits (e.g., heatstroke mice had hypothermia when exposed to room temperature), and hypothalamic ischemia, inflammation, oxidative damage, and neuronal damage, and, therefore, hypothalamic HSP72 overexpression appears to be critical to the development of thermotolerance and protection from heat-induced multiple organ dysfunction or failure.

When the mice were exposed to heat stress at 38.5°C for 60 min, FOS-positive neurons increased in the hypothalamic areas, which were thought to be involved in thermoregulation, fluid regulation, and neuroendocrine effector systems [28]. Food consumption and body weight gains decreased after a daily 60 min exposure to 38.5°C for 2 weeks [29]. The corticosterone and vasopressin levels in the blood, and catecholamine and serotonin metabolite levels in the hypothalamus augmented when above 37°C for 60 min. Especially, exposure to 38.5°C 85% RH for 60 min caused low corticosterone levels [29], which were believed to be affected in heat-induced animal death.

To our knowledge, no relevant data differentiate behavioral biochemical differences between the heat stress [-] and [+]HSP72 groups. For example, did the [-]HSP72 animals exhibit a longer recovery time after heat stress so that they stayed unconscious at the time when the body temperature was measured 3 hours after the heat stress treatment terminated? Did either group display different patterns of drinking or eating after heat stress? Did the groups demonstrate differences in body weight regulation? Prospectively, it would be very interesting for investigating these mice in metabolic chambers.

## Conclusion

In summary, when exposed the mice that contain a transgene for the porcine heat shock protein 70 gene to heat stress, the transgenic mice exhibited extraordinary responses, including the ability to survive the stress. A large number of indicators demonstrate that neural samples of the hypothalamus from the surviving mice showed better adaptation. The group suggests that injury of neural tissue of the hypothalamus is related to the inability of normal mice to withstand heat stress, and that the overexpression of the HSP70 transgene reduced hypothalamic damage from the stress, permitting the transgenic animal better adaptation.

## Methods

### Mice

Transgenic mice that were heterozygous for a porcine HSP72 gene ([+]HSP72) and transgene-negative littermate controls ([-]HSP72) were obtained from Dr. W.C. Lee at Animal Technology Institute Taiwan (ATIT, Chunan, Miaoli, Taiwan) [30]. Two groups of mice, each weighing 29 ± 0.6 g (~9 wk of age), were used. Groups of four mice were housed separately in a group in Nalgene polycarbonate cages (46 cm × 24 cm × 20 cm) that had been fitted with HEPA filter cage tops and wood-chip bedding. Rodent laboratory chow and water were provided *ad libitum *as mice were acclimated to the ambient temperature (Ta) of 26 ± 2°C for a minimum of 1 wk before experimentation (12:12-h light/dark cycle; lights on at 0600). All protocols were approved by the Animal Ethics Committee of the Chi Mei Medical Center (Tainan, Taiwan) in accordance with the guide for the Care and Use of Laboratory Animals of the National Institutes of Health as well as the guidelines of the Animal Welfare Act. In total, 96 mice were used in the present study.

### Murine model of heatstroke

Institute of Cancer research male mice 8 to 10 weeks old were exposed to heat stress treatment (42.4°C; relative humidity, 50%~55%; 1 h) in an environment-controlled chamber [2]. The heat-stressed mice were returned to the normal room temperature (26°C) after the end of the heat exposure. Mice that survived to day 4 of heat treatment were considered survivors, and the data were used for analysis of the results. Core temperatures were measured every 1 min with a copper constant thermocouple inserted into the rectum and connected to a thermometer (HR1300; Yokogawa, Tokyo, Japan). Before the start of thermal experiments, mice were housed at an ambient temperature (26°C) below the thermoneutral zone for this species [31]. After the 1 h heating period, animals were properly fed and hydrated. When unanesthetized mice were subjected to acute heat stress by exposing them to whole body hyperthermia (WBH) treatment, the stress response indicators, such as mortality, hypothermia, neuronal damage, and the levels of inflammatory cytokines and cellular ischemia and damage markers in hypothalamus were observed.

### Construction of plasmid vector and generation of transgenic mice

The procedures for generating transgenic mice were those described elsewhere in a previous report [30]. Transgenic mice were generated using a chimeric transgene ([+]HSP70) that consisted of a porcine HSP70.2 gene (Genbank accession no. AY466608) inserted into vector pCx-EGFP. The pCX-EGFP construct places the porcine HSP70.2 gene under the control of the β-actin promoter (PACTIN) and a reporter V5 gene in carboxy terminal. The chimeric transgene was cut from the plasmid by SalI and HindI digestion, purified, and used to generate transgenic mice. The linear DNA preparation was diluted in TE buffer (10 mM Tris·HCl, 0.1 mM EDTA, pH7.4) to 2 μg/μl. DNA fragments were micro injected into the pronuclei of fertilized eggs to produce transgenic mice. Prepubertal breed ICR mice were used as embryo donors and recipients. The genomic DNA of transgenic positive mice was screened by performing the PCR with primers F8:5'-GAC GCC AAC GGC ATC CTG AAC-3' and HSP70 V5R: 5'-GCC GAA TTC CGT AGA ATC GAG ACC GAG-3'. The PCR reaction was conducted at 95°C for 45 s; 58°C for 45 s, and 72°C for 45 s, repeated for 35 cycles. The length of the PCR product was 0.6 kb. F2 transgenic mice were used to proceed all experimentation. A total of 280 fertilized eggs that contained pACTIN-HSP70-V5 transgene were impregnated in 10 foster mothers by pronuclear microinjection. Of these porcine HSP70.2 recipients, seven foster mothers were successfully implanted and a total of 58 pups were obtained. Transgenic and nontransgenic mice were screened by PCR as stated above. One male pup was verified as carrying the PACTIN-HSP70-V5 transgene. The ICR mouse was served as the control.

### HSP72 detection

The animals were killed by decapitation at the end of the experiment for detection of HSP72. The hypothalamus was quickly dissected from the brain and placed into microcentrifuge tubes, and then stored at -20°C. For protein extraction, the samples were weighed, rapidly thawed in 5 volumes of homogenizing buffer consisting of 0.3 M sucrose, 50 mM Tris-HCl, and 0.17 mM Pefabloc S.C., and then homogenized by a sonic Vibra Cell (Sonic and Material Co., CT USA). After centrifugation at 13,200 × g for 5 min at 4°C, total protein concentrations were analyzed using a Bio-Rad protein asay kit (Bio-Rad, Hercules, CA, USA). The samples (40 mg/lane) were incubated for 5 min at 4°C, total protein concentrations were analyzed using a Bio-Rad protein assay kit (Bio-Rad, Hercules, CA, USA). The samples (40 mg/lane) were incubated for 5 min at 95°C in Laemmli buffer and then separated on 10% SDS-polyacrylamide discontinuous gel. After electrophoresis, the gels were processed for an immunoblotting study that was performed as described previously [27]. Polyclonal antibody specific for HSP72 (SPA-812, Stress Gen Canada) and monoclonal antibody specific for actin (CB-111, Cashmere-Biotech, USA) were used as the primary antibodies, and then anti rabbit and the antimouse immunoglobulin G conjugated with horseradish peroxidase (Amersham Pharmacia, UK) were used as the secondary antibodies. The membranes were washed and then incubated in chemiluminescence reagent plus kit (New England Nuclear Life Science Products, boston, MA, USA) and exposed to x-ray film. Expression of HSP72 and actin were semiquantified using a gel densitometric scanning program.

### Extracellular levels of glutamate, lactate-to-pyruvate ratio, glycerol, nitric oxide, and hydroxyl radicals in hypothalamus

The nitric oxide (NO_x_) concentration in the dialysates of hypothalamus were measured with the Eicom ENO-20 NO_x_^- ^analysis system (Eicom, Kyoto) [32]. In the Eicom ENO-20 NO_x_^- ^analysis system, after the NO_2_^- ^and NO_3_^- ^in the sample have been separated by the column, the NO_2_^- ^reacts in the acidic solution with the primary aromatic amine to produce an azo compound. Following this, the addition of aromatic amines to the azo compound results in a coupling that produces a diazo compound and the absorbance rate of the red color in this compound is then measured. For measurement of glutamate, lactate-to-pyruvate ratio, and glycerol, the dialysates were injected onto a CMA600 microdialysis analyzer (Carnegie Medicine, Stockholm, Sweden). The concentrations of hydroxyl radicals were measured by a modified procedure based on the hydroxylation of sodium salicylate by hydroxyl radicals, leading to the production of 2,3-dihydroxybenzoic acid (2,3-DHBA) and 2,5-DHBA [33]. A Ringer solution containing 0.5 mmol/L sodium salicylate was perfused through the microdialysis probe at a constant flow rate (1.2 mL/min). After 2 h of stabilization, the dialysates from the samples were collected. An Alltima reverse-phase C18 column (Bioanalytical System, Lafayett, Ind. 150 × 1 mm inside diameter, particle size 5 mm) was used to separate the DHBAs, and the mobile phase consisted of a mixture of 0.1 M chloroacetic acid, 26.87 nmol/L disodium EDTA, 688.76 nmol/L sodium octyl sulfate, and 10% acetonitrite (pH3.0). The retention time of 2,3-DHBA and 2,5-DHBA were 8.1 and 6.0 min.

### Determination of lipid peroxidation

Lipid peroxidation was assessed by measuring the levels of malondialdehyde (MDA) with 2-thiobarbituric acid (TBA) to form a chromophore absorbing at 532 nm [4]. About 0.1 g of tissue was homogenized with 1.5 mL of 0.1 M phosphate buffer at pH3.5. The reaction mixture (0.2 mL of sample, 1.5 mL of 20% acetic acid, 0.2 mL of 8.1% sodium dodecyl sulfate, and 1.5 mL of aqueous solution of 0.8% TBA, up to 4 mL with distilled water) was heated to 95°C for 1 h, and then 5 mL of N-butanol and pyridine (15:1 vol/vol) was added. The mixture was vortexed vigorously, centrifuged at 1500 g for 10 min, and the absorbance of the organic phase was measured at 532 nm. The values were expressed as nanomoles of TBA-reactive substances (MDA equivalent) per milligram of protein.

### Quantification of total and oxidized glutathione

Tissues were homogenized in 5% 5-sulfosalicylic acid (1:10 wt/vol) at 0°C, and the supernatants were used for analysis of total and oxidized glutathione. Total glutathione [reduced-form glutathione (GSH) + oxidized-form glutathione (GSSG)] was analyzed according to the Tietze method [34], and GSSG was determined as described by Griffith [35]. The recycling assay for total glutathione is oxidized by 5,5-Dithiobis [2 acid] (DTNB) to give GSSG with stoichiometric formation of 5-thio-2-nitro-benzoic acid. GSSG is reduced to GSH by the action of the highly specific glutathione reductase (GR) and nicotinamide adenine dinucleotide phosphate (reduced form; NADPH). The rate of 5-thio-2-nitro-benzoic acid formation is followed at 412 nm and is proportional to the sum of GSH and GSSG present.

### Determination of glutathione peroxidase (GPx) and glutathione reductase (GR) activity

To measure cytosolic GP_x _and GR activities tissues were homogenized in 0.05 M phosphate buffer, pH7.0. The homogenates were centrifuged at 4000 × g for 20 min at 4°C, and supernatants were used for GP_x _and GR activity assay. The GP_x _and GR activities were performed with a commercial glutathione peroxidase activity assay kit (Sigma, USA) and a GR assay kit (Sigma, USA), respectively. One unit of GP_x _and GR activity was defined as the amount of sample required to oxidize 1 mmol of NADPH per minute based on the molecular absorbance of 6.22 × 10^6 ^for NADPH.

### Determination of ATP

The hypothalamic sample was frozen in liquid nitrogen. Frozen tissues were transferred to a 1.5 mL microfuge tube, and ice-cold 0.5 N perchloric acid, 10 μL/mg wet weight, was added. The tissue was immediately homogenized with a sonic Vibra Cell (Sonic and Material Co., CT, USA). The acidic homogenate was centrifuged at 9200 × g for 5 min at 4°C. The supernatant was neutralized with 3 M potassium hydroxide, kept on ice for 10 min to promote precipitation of the perchlorate, and then centrifuged again. A 500 mL portion of the supernatant was added to a mixture of 50 μl of chloroacetaldehyde reagent and 450 mL of dipotassium hydrogen phosphate. The samples were then incubated at 80°C for 40 min. The reaction was terminated by placing on ice and then stored at -80°C until HPLC assay.

### Neuronal damage score

At the end of each experiment the brain was removed, fixed in 10% neutral buffered formalin, and embedded in paraffin blocks. Serial (10 μm) sections through the hypothalamus were stained with hematoxylin and eosin for microscopic evaluation. The extent of hypothalamic neuronal damage was scored on a scale of 0-3, modified from the grading system of Pulsinelli et al. [36], in which 0 is normal, 1 means that ~30% of the neurons are damaged, 2 means that ~60% of the neurons are damaged, and 3 means that 100% of that neurons are damaged. Each sample was evaluated independently without knowledge of the experimental conditions.

### Determination of cytokines in the hypothalamus

For determination of tumor necrosis factor-α (TNF-α), interleukin-1β (IL-1β), and interleukin-10 (IL-10), brains were taken 4 h after the start of heat stress. The brain (hypothalamus) samples were prepared according to previous reports [37]. The tissues were homogenized in five volumes of ice-cold Ripa buffer. The homogenates were incubated on ice for 30 min and then centrifuged (15,000 × g, 30 min, 4°C) twice. The supernatants were stored at -70°C until time of measurement. The concentrations of these cytokines in tissue lysates were determined using double-antibody Sandwich ELISA (R & D Systems, Minneapolis, MN, USA) according to the manufacture's instructions. Optical densities were read on a plate reader Set at 450 nm for these cytokines. The concentrations of these cytokines in the samples were calculated from the standard curve multiplied by the dilution factor and was expressed as pg/g.

### Data presentation and statistical analysis

Data are presented as mean ± SEM. ANOVA was used to conduct factorial experiments, and Duncan's multiple-range test was performed to make *post hoc *multiple comparisons among means. The Wilcoxon's signed rank test was used to compare the neuronal damage across two groups. The Wilcoxon tests convert scores to ranks, a sum of the rank is calculated, and critical values of the sum are provided to test the null hypothesis at a given significant level. The data are given as "median", first quartile, and third quartile. A *P *value of less than 0.05 was considered as statistically significant.

## Abbreviations

HSP: heat shock protein; [+]HSP72: transgene-positive HSP72; [-]HSP72: transgene-negative HSP72; DHBA: dihydroxybenzoic acid; IL-1β: interleukin-1β; TNF-α: tumor necrosis factor-α; IL-10: interleukin-10; MDA: malondialdehyde; GSSG: oxidized-form glutathione; GSH: reduced-form glutathione; GR: glutathione reductase; GPx: glutathione peroxidase; WBH: whole body hyperthermia.

## Authors' contributions

ZCC performed heat treatment, physiologic assessment, data analyses and co-wrote the manuscript. WSW contributed to generation of transgenic mice and biochemical assessment. MTL and CCH participated in the development and supervision of the overall research design and protocols and co-wrote the manuscript.
